# Dynamic Soluble IL-6R/Soluble gp130 Ratio as a Potential Indicator for the Prostate Malignancy Phenotype—A Multicenter Case–Control Study

**DOI:** 10.3390/jpm14101037

**Published:** 2024-09-28

**Authors:** Cosmin-Victor Ene, Bogdan Geavlete, Cristian Mares, Ilinca Nicolae, Corina Daniela Ene

**Affiliations:** 1 Department of Urology, ‘Carol Davila’ University of Medicine and Pharmacy, 050474 Bucharest, Romania; bogdan_geavlete@yahoo.com (B.G.);; 2 Department of Urology, “Saint John” Clinical Emergency Hospital, 042122 Bucharest, Romania; 3 Department of Research, ‘Victor Babes’ Clinical Hospital for Infectious Diseases, 030303 Bucharest, Romania; 4 Department of Nephrology, ‘Carol Davila’ Nephrology Hospital, 010731 Bucharest, Romania; corina.ene@umfcd.ro; 5 Department of Nephrology, ‘Carol Davila’ University of Medicine and Pharmacy, 020021 Bucharest, Romania

**Keywords:** prostate tumors, microenvironment, inflammation, IL-6 signaling, acute phase reaction, oxidative stress

## Abstract

Objective: Prostate tumors, if prostate cancer or adenoma, represent a major public health challenge. Progress in research on inflammation has revealed a connection between inflammation, immunity, and cancer. In this context, this study aimed to find IL-6 signaling systemic abnormalities in the inflammatory tumor microenvironment. Material and methods: This study was case–controlled, multicentered, and included 86 patients, 43 diagnosed with BPH and 43 diagnosed with PCa, between January 2019 and January 2020. The study group was homogenous and the studied parameters were IL-6 complex (IL-6, soluble receptor IL-6R, soluble glycoprotein gp130), acute phase proteins (C reactive protein—CRP, acid alpha1 glycoprotein—AGPA, ferritin, albumin, transferrin), and oxidative stress-associated variables (malondialdehyde—MDA, carbonylated protein—PCO, 8-hydroxy-deoxy guanosine-8-OHdG, total antioxidant status—bTAS). Results: The inflammatory microenvironment determined IL-6 signaling alterations (over-regulation of sIL-6R and suppression of sgp130 in PCa versus BPH), changes in acute phase reaction markers (increased serum levels of CRP, AGPA, ferritin, and decreased serum levels of albumin, transferrin) that were much more evident in PCa compared to BPH, an imbalance between macromolecular oxidative damage (MDA, PCO, 8-OHdG) and endogenous antioxidants (TAS) that was more accentuated in PCa compared with BPH, and a representative association between the sIL-6R/sgp130 ratio and inflammatory/oxidative stress-related factors only in PCa patients. Conclusions: Our study reconfirms the anterior concept that IL-6 promotes prostatic tumorigenesis. In this study, we first demonstrated that a high sIL-6R/sgp130 ratio facilitates prostate malignancy.

## 1. Introduction

Prostate tumors, if prostate cancer or adenoma, represent a serious public health challenge because the lifetime risk of a man developing histologically confirmed benign prostate hyperplasia (BPH) has been reported to be 70% in men in the 61–70 years age group [1], while prostate cancer (PCa) has been proven to be the first type of cancer in men after 66 years [2]. However, the exact etiology of both pathologies has not yet been elucidated.

**Figure 1:**
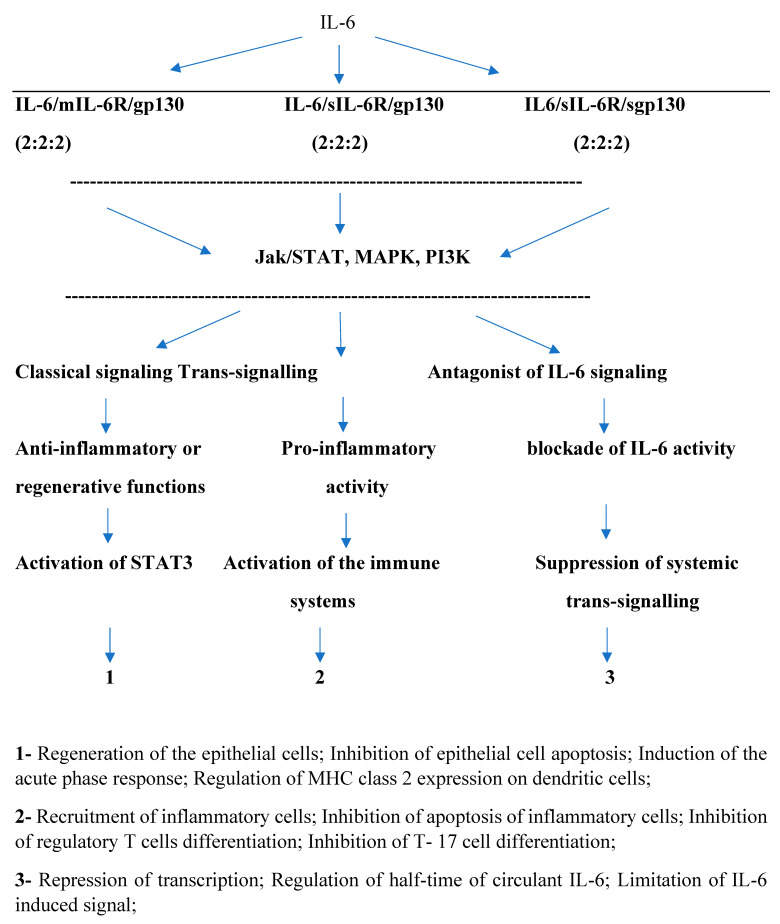
Schematic illustration of IL-6 signaling IL-interleukin; IL-6R-IL6 receptor; mIL-6R-membranar-IL-6R; gp130-glycoprotein; sIL-6R-soluble IL-6R; sgp130-soluble; gp130; jak/STAT-Janus kinase/signal transducers and activators of transcription; MAPK-Mitogen-activated protein kinase; and PI3K- Phosphoinositide 3-kinase.

The interplay between inflammation, immunity, and tumor microenvironment in prostate tumors demonstrates that IL-6 production might influence tumor growth. Recent studies have validated that the bidirectional relation between tumor cells and microenvironment produces tumor growth and inhibits tumor cell clearance by the immune system [3]. Moreover, it was observed that the tumor microenvironment acquires many characteristics. In this way, fibroblasts become tumor-associated fibroblasts, macrophages promote cytokine secretion, myeloid-derived suppressor cells migrate from bone marrow to the tumor, where they transform themselves into stromal cells, and adipocytes produce tumor factors that contribute to invasion. Also, tumor-infiltrating immune cells contribute to immune evasion. The microenvironment acts like a biological barrier that protects the tumor from the immunological and nonimmunological damage of antitumoral medication aggression [3,4,5]. Furthermore, the dynamic process of macrophage polarization defines the pro-inflammatory and anti-inflammatory phenotype [6]. The inflammatory microenvironment, microbiome, androgen/androgen receptors status, hypoxia, and genomic instability promote neoplastic transformation via oxidative stress [7,8,9]. Systemic cytokines (IL-6, TNFalpha, IL-10, TGFbeta) and chemokines (CCL2, CCL4, CXCL10) from the environment provide cell-to-cell communication in the regulation of inflammation [3,8,10]. IL-6 is implicated in the initiation, development, and end of inflammatory response. The most important sources of IL-6 are represented by macrophages, B and T lymphocytes, fibroblasts, endothelial cells, and tumor cells. In the tumor microenvironment, IL-6 mediates pro-inflammatory and anti-inflammatory effects via classic signaling [3,5,7,8,11,12] [Figure 1].

In this context, inflammation seems to be the key element in prostate tumors because large population-based trials such as the Medical Therapy of Prostate Symptoms (MTOP) and, more recently, the Reduction by Dutasteride of Prostate Cancer Events (REDUCE) studies demonstrated the association between inflammation and the development of BPH [13,14], while chronic inflammation is thought to be one of the numerous driving factors for the development of PCa [15]. The most famous marker in inflammation, with a certain contribution to cancer development, is CRP [16]. In PCa, the high levels of CRP in association with IL-6 are correlated with a lower survival rate, no matter what the PSA values are [17]. Another parameter with a potentially high relevance is the alpha-1-acid glycoprotein (AGP-1), named orosomucoid (ORM). ORM is an acute-phase reaction protein that is supposed to play an important role in the tumor microenvironment through its influence on the immune system, drug resistance, cancer progression, and metastasis [18]. This reactant has a higher expression in prostate carcinoma, while its levels are variable in different types of cancer. These studies also showed that the serum hs-CRP level in the prostate cancer group was higher than that of the BPH group [19].

Another relevant parameter which plays a pivotal role both in developing PCa and BPH is represented by oxidative stress (OS) [15]. In this context, it is important to mention malondialdehyde (MDA) as a marker to reflect oxidative status in normal subjects, BPH and PCa patients [20,21,22], near the carbonylated proteins, which are less studied but play a similar role.

IL-6 has been shown to have a role in the development and progression of prostate cancer in both in vivo and in vitro studies [23]. Soluble gp130 is a regulator of interleukin-6/soluble interleukin-6 receptor signaling that influences prostate cancer in an interleukin-6-dependent and independent manner. It may also regulate maspin expression in the absence of the IL-6, confirming the already proposed binding of sgp130 to other ligands [24].

During chronic inflammation, some cytokines regulate the inflammatory response by cell-to-cell signaling. Bearing in mind all the above-mentioned aspects, this study aimed to investigate the IL 6 signaling response to the inflammatory prostate microenvironment.

## 2. Material and Methods

### 2.1. The Characteristics of Groups

This study was developed at St. John Clinical Hospital of Emergency and was prospective and multicenter. At the beginning of the study, 141 patients were screened, but only 86 patients accomplished the inclusion criteria (aged over 45 years, without other neoplasia, without inflammatory diseases, without anti-inflammatory treatment, or normal nutritional status): 43 were diagnosed with BPH, and 43 were diagnosed with PCa using prostate transrectal biopsy during January 2019 and January 2020. The study groups were homogenous, and all the patients were included after signing an informed consent form approved by the ethical committee according to the Helsinki Declaration. Patients included in the study did not receive treatment for prostatic pathology before beginning the study. The patients with prostatic cancer did not present metastasis on a thoracic-abdominal–pelvic CT scan nor locally advanced disease. The maximal Gleason score for the included patients was 7 (3 + 4).

### 2.2. Biologic Samples: Ethics Statement

The biologic samples were collected before the prostate biopsy for suspect patients and were kept conserved at −180 degrees until the biopsy and the CT scan confirmed the patient’s eligibility. For the patients with BPH who were diagnosed using a digital rectal examination, PSA, free PSA, and ultrasonography, the samples were processed immediately after collecting them, a jeun. All the icteric, hemolyzed, milky, or microbiologic-contaminated samples were eliminated from the study (n = 52 samples). The samples for laboratory determinations were collected after signing the informed consent form.

### 2.3. Laboratory Tests

Interleukin-6 was measured using the ELISA-sandwich technique (EnzoLife reactive TECAN analyzer, Bio-Connect, The Netherlands). The sensitivity of the method was 0.057 pg/mL, and the assay range was 1.56–50 pg/mL. Soluble IL-6R and soluble gp130 were determined by the solid phase sandwich ELISA method using an immune-enzymatic kit (RD Systems, Minneapolis, MN, USA, Soluble IL-6R by DR600 kit) with an analytical sensitivity of 15.1 pg/mL and an assay range of 31.2–2.000 pg/mL. Soluble gp130 was evaluated using DGP00, with an analytical sensitivity of 0.08 ng/mL and an assay range of 0.1–8 ng/mL. The results were evaluated at 450nm using a TECAN analyzer (TECAN, Switzerland). hs-CRP, AGPA, ferritin, albumin, and transferrin were evaluated by immunoturbidimetry based on the agglutination reaction of latex microparticles coated in antigen/antibody molecules and the test serum using human reactants kits. The results were quantified by HumaStar300 analyzer (HUMAN Gesellschaft from Biochemical and Diagnostics GmbH, Wiesbaden, Germany). Malondialdehyde, a peroxidation marker of membrane phospholipids, forms a complex with thiobarbituric acid that is measured spectrophotometrically. Biological samples contain a mixture of substances that react with thiobarbituric acid (TBARS), which includes lipid hydroperoxides and aldehydes. In practice, TBARS is expressed by the MDA value. The absorbance at 532 nm is read (Sigma reagent, BS3000 analyzer, SINNOWA Medical Science and Technology, Nanjing, China). The results are expressed in nmol/mL serum.

Carbonylated proteins, an indicator of oxidative stress in biological systems, react with 2,4-dinitrophenylhydrazine (DNPH) to form the corresponding hydrazone, which is measured spectrophotometrically (BS3000 analyzer) and with the Merck reactive (MAK094kit, Merk Company, Darmstadt, Germany). The absorbance at 370 nm from the blank is read. The carbonylated protein content is expressed in µmol/L. For 8 hydroxy-deoxy-guanosine (8-OH dG), an oxidative DNA damage ELISA kit was used (immunodiagnostic reagent, TECAN analyzer).

The total antioxidant status (TAS) was determined with the spectrophotometric method using the RANDOX kit (Randox Laboratory Ltd., Crumlin, UK). ABTS (2,2-azino-di-3-ethylbenzthiazoline sulfonate) is incubated with a peroxidase (metmyoglobin) and hydrogen peroxide to produce the cationic radical ABTS. It has a stable blue-green color that is measured at 600 nm (Huma Star300 analyzer, Human, Germany). The antioxidants in the sample induce the suppression of color production at a level proportional to their concentration.

### 2.4. Statistical Analysis

All the data were analyzed using the Pearson coefficient in the SPSS 19 statistical program.

The results are presented as the mean and standard deviation. Data comparison was performed using either ANOVA for normally distributed data with Tukey’s post hoc test or Kruskal–Wallis test or for non-normally distributed data using Dunn’s post hoc test. Pearson’s correlation coefficient was used to evaluate the relation between the parameters. The Kolmogorov–Smirnov test was used to evaluate data normality before the assessment. To test the hypothesis, 0.05 (5%) was the chosen level of significance, and 95% was the confidence interval.

## 3. Results

There was a group of 86 patients, equally divided into two groups, with 43 cases in each group. The arms were homogenous, with similar general characteristics, such as age, BMI, the value of glycemia, Hb level, creatinine, and uric acid. The general parameters that were modified were prostate volume (higher volume in the BPH group), PSA and free PSA, the IPSS score, and alkaline phosphatase (higher volume in the PCa group) [Table 1].

The analysis of IL-6 signaling system alterations in prostatic tumor patients is presented in Table 2. We detected an IL-6 increase of 1.07-fold in PCa compared with BPH (*p* > 0.05). sIL-6R increased 1.55-fold (*p* < 0.05), while sgp 130 decreased 1.34-fold in PCa compared to the BPH group (*p* < 0.05). The sIL-6R/sgp 130 ratio was 2-fold higher in PCa compared to the BPH group (*p* < 0.05).

Concerning the inflammatory status of the studied groups, it can be observed that there was a statistically significant increase (*p* < 0.05) of 4.77-fold of CRP, respectively, 1.65-fold of ferritin and 4.77-fold of acid alpha glycoprotein in PCa compared to the BPH group. Albumin decreased by 1.16-fold (*p* < 0.05), while transferrin was 1.44-fold (*p* < 0.05) in the PCa group when compared with the BPH group (Table 3).

Regarding the level of oxidative stress in the studied arms, statistically significant differences (*p* < 0.05) were observed for all of the studied parameters (Table 4): malondialdehyde was 1.19-fold higher in the PCa group compared with the BPH group (*p* < 0.05), carbonylated proteins increased 1.51-fold (*p* < 0.05), and 8-OH dG increased 1.57-fold (*p* < 0.05), while the total antioxidant status decreased 1.85-fold in the PCa group compared to the BPH group (*p* < 0.05) [Table 4].

We analyzed the interplay between IL-6 system signaling and changes in inflammation by calculating the Pearson coefficient (Table 5). sIL-6R and sIL-6R/sgp 130 correlated strongly positively, while sgp130 correlated strongly negative with CRP (*p* < 0.05) in the PCa group. sgp130 and sIL-6R/sgp 130 correlated strongly positively with AGPA (*p* < 0.05) in the PCa group. sgp130 correlated strongly negatively with ferritin (*p* < 0.05) in the PCa group. sgp130 correlated positively with albumin in the BPH group (*p* < 0.05), with no correlations detected in the PCa group. sIL-6R and sIL-6R/sgp 130 correlated negatively positively, while sgp130 correlated positively with transferrin in the PCa group (*p* < 0.05). Both sgp130 and sIL-6R/sgp 130 ratios correlated negatively with 8OHdG in the PCa group (*p* < 0.05). Only sgp130 correlated with TAS in the PCa group (*p* < 0.05). No relations were detected in the BPH group between the studied markers.

## 4. Discussion

In this study, we investigated the IL-6 signaling response to the inflammatory microenvironment of prostate tumors. The main point of this study is that IL-6 is overexpressed in BPH and PCa. This remark supports the anterior hypothesis that IL-6 facilitates the tumorigenesis of benign prostate epithelial cells and also metastatic progression [25]. Significant statistical differences between BPH and PCa were identified between serum values of soluble components of IL-6R. So, in the tumor microenvironment, the quantity of sIL-6R is smaller than the amount of sgp130 in BPH patients, resulting in the concomitant activation of two signaling pathways mediated by IL-6 (the classic way and trans-signaling). In PCa, the quantity of sIL-6R is greater than the quantity of sgp130, suggesting that trans-signaling overexpression is coordinated by IL-6. These outcomes support the hypothesis that the mechanisms that produce IL-6 signaling in benign and malignant prostatic tumors are seriously damaged during persistent inflammation [10]. An sIL-6R excess overexpresses trans-signaling, while an sgp130 excess reduces trans-signaling [10,26,27,28,29].

In conclusion, our results evidence the possible role of the sIL-6R/sgp130 ratio in the differential diagnosis of BPH from PCa. This observation conceptually supports that IL-6 acts both as a marker and as a mediator of inflammation [10,25]. Also, in the current paper, we observed that IL-6 modulates acute phase reaction markers and oxidative stress-related parameters in prostate tumors. IL-6 overproduction induces the synthesis of positive acute phase reactants (hsCRP, AGPA, ferritin) and the exacerbation of the oxidative degradation of organic macromolecules (nucleic acids, glycoproteins, glycolipids), and the suppression of negative acute phase reactants (albumin, transferrin) and of endogenous antioxidants in PCa patients. Recent data show that acute phase reaction reactants affect interactions between different cells acting within the microenvironment, including leucocyte profiles, inflammatory resolution, and tissue regeneration, mediating endocytosis, the lipid raft phenotype, serin protease inhibition, and macrophage polarization towards the pro-tumorigenic M2-like profile [30]. Over the last decade, the association between CAP, oxidative stress, and antioxidants in tumors has been demonstrated. Upregulated oxidative stress-related variables initiate CAP via the modulation of androgens, inflammation, vitamin D, tumor suppressor protein p53, and antioxidants [31]. Several epidemiological data have revealed that the mechanism of action of antioxidants in the inhibition of prostate carcinogenesis could include reductions in DNA damage, the inhibition of cell growth and division via the generation of reactive oxygen species, a reduction in the expression of the surviving gene and epigenetic modulators, the stimulation of apoptosis, and regulation of PI3K/Akt, NFKb, Akt/mTOR cascade, and androgen receptor signaling [32]. In this study, in patients with BPH and PCa, there was evidence of the over-regulation of growth factors, increasing oxidative stress, and amplification of inflammation. Oxidative stress and inflammation are two interconnected processes in prostatic pathology. Inflammation takes part in the immune response, while oxidative stress is implicated in promoting and developing chronic inflammatory processes. Reactive oxygen species produce the oxidation of biomolecules and amplify the inflammation [33].

When comparing the studied groups, it can be observed that in the case of CRP and of acid alpha glycoprotein, there were higher levels for the PCa arm, while the level of IL-6 did not change from one pathology to another. This aspect was noted in another comparative study from the literature for CRP [19], which is considered an independent predicting factor of cancer-specific survival in prostate cancer patients [34] near AGP-1 and is also associated with cancer progression [35]. However, the effects of these parameters in BPH, where an important increase was associated with advanced symptoms, are not very clear [36]. For IL-6, which is also implicated in the tumor process, Adler et al. [37] reported that patients with metastatic PCa had significantly elevated IL-6 levels when compared with those in other PCa groups, explaining the relatively normal value in our group, bearing in mind that the enrolled patients were metastasis-free, with low-level cancer. As is known from the literature, IL-6 is involved in the regulation of various cellular functions, including proliferation, apoptosis, angiogenesis, differentiation, and regulation of immune response [38]. Although IL6 levels did not vary significantly between our groups, we found a very interesting positive correlation between the IL6 serum level and prostate volume. This is the first study in the literature that shows such a significant correlation. IL6 is known as a marker of metastasis in PCa subjects, but in prostate hypertrophy, our result could be explained by the high production of IL6 in prostate tissue by epithelial basal cells, as demonstrated by immune-histologic determinations [39]. The level of oxidative stress in the studied arms was characterized by an increased level of malondialdehyde, carbonylated proteins, 8-OHdG, and total antioxidant status for the PCa group when compared with the BPH group, but this is controversial in the literature. Some studies support our outcomes, like Arsova-Sarafinovska et al. [20], but Kucukdurmaz et al., in a comparative study on 81 cases, found that MDA concentrations were insignificantly increased in all PCa groups when compared to the BPH group [40]. We found some interesting negative correlations between prostate volume and carbonylated proteins, respectively, on total antioxidant status that have not been mentioned in the literature before. In our study, the alteration of the balance between prooxidants and antioxidants was proved by the increasing oxidative degradation of proteins, lipids, and nucleic acids and the decreasing antioxidant capacity. Antioxidants could modulate some signal pathways, which resulted in increasing the expression of some redox-sensitive transcription factors (Nrf2, Nf-kb, AP1, and MAPK) and the overproduction of some proinflammatory cytokines [41,42,43]. The modulation of those factors changed the oxidative stress signaling pathways implied in prostate pathology. Even though there are a lot of working hypotheses, oxidative stress measurements in BPH and PCa remain a challenge due to the volatility of the compounds and the variability of different involved signaling pathways and mechanisms. In this context, although strong correlations are seen between oxidative stress and prostate cancer, clinical translations are lacking [44]. 8-OHdG is the most frequently detected and studied DNA lesion, which is now considered a biomarker of generalized, cellular oxidative stress and is linked to degenerative diseases, including cancer [45]. Pande et al. found that with an increase in Gleason score > 6, the 8-OHdG level raised significantly [46], while Chiou et al. remarked that the competitive ELISA for 8-OHdG and its analogs appears to be a simple method for quantifying the extent of oxidative stress and may have the potential for identifying cancer risk [45].

Our results suggest that the high production of proinflammatory cytokines and increased oxidative stress in prostate pathology might be associated with the high production of PSA [47,48]. This study is the first in the literature to evaluate a large panel of inflammatory, oxidative stress, and angiogenic markers in main prostate tumors—cancer and hypertrophy, which seems to be useful in clinical practice and highlights a common pathway of these pathologies and some specific characteristics. This could be the beginning point for further study in the literature to find a panel list of serum markers other than PSA. The sIL-6R/sgp130 ratio could be evaluated as a differential diagnostic tool between BPH and PCa in relation to PSA as urologists continue the quest for the ideal tumor marker, but further studies will be needed [49]. Our study has some limitations, like the short period of follow-up of the subjects, the lack of a control group, and the determination of these markers only at baseline. The lack of a study strength assessment is also a study limitation. Further studies are needed to evaluate the levels of these molecules over time and after surgical removal of the tumor in the PCa group. It would be worthwhile in the future to determine whether the sgp130, sIL-6R, and IL-6 plasma levels are altered in PC patients with a worse prognosis because these data could identify those patients who could benefit from anti-cytokine therapy.

## 5. Conclusions

BPH and PCa pathophysiology is currently incompletely understood, although their evolution could have some common pathways. However, the results of our study show abnormal circulant levels of antioxidant and angiogenesis factors in patients with BPH and PCa, validating the hypothesis that prostatic tumorigenesis is correlated with the over-regulation of oxidative stress and inflammation, concomitant with a better understanding of the biologic mechanism. Assessing the role of studied biomarkers seems to be useful in clinical practice to anticipate the evolution of the prostate pathology and find the best therapeutic approach. IL-6, a major cytokine in this microenvironment near chronic inflammation and oxidative stress, plays a key role in tumorigenesis. In this study, for the first time, it was demonstrated that a high sIL-6R/sgp130 ratio facilitates prostate malignancy. Selective IL-6 signaling blockade could be explored in the future as a valid candidate for the treatment of BPH and CaP.

## Figures and Tables

**Table 1:** General parameters of the studied groups. Clinicopathological features of the patients.

Group’s Characteristics	BPH Patients (43 Cases)	PCa Patients (43 Cases)	Statistical Significance
Age (years)	68 ± 11	66 ± 15	*p* = 0.21
Prostate volume (cm^3^)	50 ± 15	39 ± 12	***p* = 0.16**
BMI (kg/m^2^)	27 ± 6	29 ± 5	*p* = 0.53
Glycemia (mg/dL)	101 ± 12	104 ± 9	*p* = 0.34
Hb level (g/dL)	13.4 ± 1.1	13.1 ± 0.9	*p* = 0.71
Creatinine (mg/dL)	0.8 ± 0.3	0.9 ± 0.2	*p* = 0.12
Urea (mg/dL)	23 ± 6	22 ± 7	*p* = 0.27
PSA (ng/mL)	2.32 ± 1.34	11.81 ± 3.89	***p* = 0.0001**
free PSA (ng/mL)	0.25 ± 0.05	0.38 ± 0.04	***p* = 0.01**
IPSS	18 ± 4	17 ± 3	***p* = 0.05**
Alcaline phosphatase (UI/L)	84 ± 34	102 ± 29	***p* = 0.01**
NLR	2.79 ± 1.46	2.88 ± 1.29	**0.154**

NLR-neutrophile lymphocyte ratio (reference control interval—1.61 ± 1.07). In the literature, NLR is used as an indicator for systemic inflammation.

**Table 2:** The IL-6, sIL-6R, and sgp130 serum levels of the studied groups. IL-6 signaling system alterations in prostatic tumor patients.

Parameter	BPH (48 Cases)	PCa (45 Cases)	*p* Value
IL-6 (pg/mL)	18.0 ± 8.3	19.4 ± 7.1	0.078
sIL-6R (ng/mL)	147.2 ± 57.1	228.5 ± 75.8	0.007
sgp 130 (ng/mL)	291.3 ± 73.5	217.4 ± 68.1	0.019
sIL-6R/sgp 130	0.51 ± 0.11	1.05 ± 0.14	0.001

BPH—benign prostate hyperplasia; PCa—prostate cancer; IL-6-interleukin (reference control interval: 3.88 ± 0.61 pg/mL ser); sIL-6R—soluble receptor (reference control interval: 107.1 ± 7.0 ng/mL ser); sgp 130—soluble glycoprotein (reference control interval: 313.1 ± 49.1 ng/mL ser); sIL-6R/sgp 130 ratio (reference control interval: 0.34 ± 0.04); and *p*-statistical significance.

**Table 3:** Acute phase protein serum levels in studied groups. IL-6 promotes acute phase response.

Parameter	BPH Patients (43 Cases)	PCa Patients (43 Cases)	*p* Value
hsCRP (mg/dL)	1.07 ± 0.38	5.11 ± 1.27	**0.013**
Ferritin (ng/mL)	97.1 ± 28.3	161.1 ± 42.6	**0.006**
Albumine (g/dL)	3.6 ± 0.6	3.1 ± 0.9	**0.012**
Transferrin (mg/dL)	207.1 ± 36.8	143.6 ± 50.6	**0.007**
AGPA (g/L)	2.39 ± 0.43	11.41 ± 6.62	**0.04**

BPH—benign prostatic hyperplasia; PCa—prostate cancer; hsCRP—high-sensitivity C-reactive protein (reference control interval: 0.16 ± 0.16 mg/dL); AGPA-alpha1 glycoprotein acid (reference control interval: 0.86 ± 0.13 g/L); ferritin (reference control interval: 47.1 ± 8.1 ng/mL); albumin (reference control interval: 4.1 ± 0.4 g/dL); transferrin (reference control interval: 225.3 ± 24.2 mg/dL); and *p*-statistical significance.

**Table 4:** The level of oxidative stress in the studied groups. IL-6 is linked to oxidative stress.

Parameter	BPH Patients (43 Cases)	PCa Patients (43 Cases)	*p* Value
MDA (mmol/mL)	3.23 ± 0.40	3.89 ± 1.30	**0.04**
PCO (umol/L)	29.10 ± 4.41	44.2 ± 6.58	**0.02**
8-OH dG (ng/mL)	6.41 ± 1.87	10.18 ± 4.13	**0.03**
TAS (mmol/L)	1.28 ± 0.23	0.69 ± 0.41	**0.01**

BPH—benign prostatic hyperplasia; PCa—prostate cancer; MDA-malonyl dialdehyde (reference control interval: 1.97 ± 0.15 mmol/mL); PCO-carbonylated proteins (reference control interval: 23.0 ± 1.8 µmol/L); 8-OHdG—hydroxy-deoxyguanosine (reference control interval: 3.11: ± 0.3 ng/mL); and TAS—total antioxidant status (reference control interval: 1.9 ± 0.3 mmol/L).

**Table 5:** Statistical correlation between IL-6 complex and the inflammation-related parameters in the studied group. The interplay between IL-6 system signaling and changes in the inflammation-associated panel.

Parameter		BPH Patients			PCa Patients		
		sIL-6R	sgp130	sIL-6R/sgp 130	sIL-6R	sgp130	sIL-6R/sgp 130
hsCRP	R	0.142	−0.107	0.112	0.283	−0.419	0.602
	*p*	0.372	0.355	0.311	**0.052**	**0.003**	**0.001**
AGPA	R	0.117	0.089	0.103	0.201	0.306	0.189
	*p*	0.701	0.325	0.835	0.510	**0.041**	**0.035**
Ferritin	R	0.151	0.061	0.10 1	0.098	−0.261	0.141
	*p*	0.221	0.655	0.531	0.821	**0.019**	0.4115
Albumin	R	0.098	0.143	0.126	−0.106	0.104	−0.030
	*p*	0.721	0.0135	0.612	0.193	0.421	0.219
Transferrin	R	−0.160	0.104	−0.085	−0.203	0.209	−0.172
	*p*	0.407	0.635	0.410	**0.050**	**0.028**	**0.051**
MDA	R	0.112	0.129	0.099	0.055	0.109	0.171
	*p*	0.432	0.415	0.995	0.950	0.525	0.2015
PCO	R	0.122	−0.066	0.022	0.087	−0.127	−0.109
	*p*	0.311	0.647	0.955	0.835	0.093	0.4195
8OHdG	R	0.098	0.048	0.083	0.198	−0.281	−0.207
	*p*	0.512	0.375	0.431	0.087	**0.025**	**0.046**
TAS	R	0.103	0.078	−0.099	−0.148	0.295	−0.152
	*p*	0.853	0.835	0.602	0.104	**0.058**	0.102

BPH—benign prostatic hyperplasia; PCa—prostate cancer; sIL-6R—soluble receptor; sgp 130—soluble glycoprotein; sIL-6R/sgp 130—ratio; hsCRP—high sensitivity C-reactive protein; AGPA—alpha1 glycoprotein acid; MDA—malonyl dialdehyde; PCO—carbonylated proteins; 8-OHdG—hydroxy-deoxyguanosine; TAS—total antioxidant status; r—correlation–coefficient; and *p*—statistical significance.

## Data Availability

The data presented in this study are available on request from the corresponding author due to the technical limitation concerning the external access to the server where they are deposed.
